# Understanding pathogenic single-nucleotide polymorphisms in multidomain proteins – studies of isolated domains are not enough

**DOI:** 10.1111/febs.12094

**Published:** 2013-01-16

**Authors:** Lucy G Randles, Gwen J S Dawes, Beth G Wensley, Annette Steward, Adrian A Nickson, Jane Clarke

**Affiliations:** Department of Chemistry, University of CambridgeUK

**Keywords:** disease-causing mutation, multidomain protein, pathogenic mutation, protein interface, single nucleotide polymorphism

## Abstract

Studying the effects of pathogenic mutations is more complex in multidomain proteins when compared with single domains: mutations occurring at domain boundaries may have a large effect on a neighbouring domain that will not be detected in a single-domain system. To demonstrate this, we present a study that utilizes well-characterized model protein domains from human spectrin to investigate the effect of disease-and non-disease-causing single point mutations occurring at the boundaries of human spectrin repeats. Our results show that mutations in the single domains have no clear correlation with stability and disease; however, when studied in a tandem model system, the disease-causing mutations are shown to disrupt stabilizing interactions that exist between domains. This results in a much larger decrease in stability than would otherwise have been predicted, and demonstrates the importance of studying such mutations in the correct protein context.

## Introduction

A key area of interest in the post-genomic era is to relate changes in gene sequence to phenotypic variation. As more than 70% of eukaryotic proteins are composed of multiple domains, when studying the effects of pathogenic mutations in multidomain proteins, we must determine the effect that a mutation in one domain may have on neighbouring domains 1. Diseases caused by missense mutations, often referred to as non-synonymous single nucleotide polymorphisms (nsSNPs), are well documented 2–4. Although some mutations directly affect an active site or binding to a ligand, most mutations affect protein function by reducing the stability of the protein 5–9. Several computational databases exist that attempt to predict the effect of nsSNPs on protein function and stability 10–13. As it is not possible to experimentally characterize the effect of all mutations on all affected proteins, we have previously shown that well-characterized model proteins may be employed to determine the effects of disease-causing mutations, a technique that is especially useful when the variant proteins are difficult to express in the laboratory 14. In this study by Randles *et al*. 14, which employed immunoglobulin-like (Ig-like) domains as models, it was found that any mutation that caused a loss of stability > 2 kcal·mol^−1^ resulted in disease. Moreover, the severity of disease correlated with the extent of destabilization. Such a ‘cut-off’ has been observed in other studies 9,15,16. In multidomain proteins in which the domains behave independently of each other (e.g. Ig domains in the I-band of titin), a mutation in one domain is highly unlikely to affect the stability of a neighbouring domain 17. However, where adjacent domains interact in multidomain proteins, the stability of one domain may be increased by interaction with its neighbours. Thus, the effect of a mutation in one domain may result in the destabilization of neighbouring domains.

**Fig 1:**
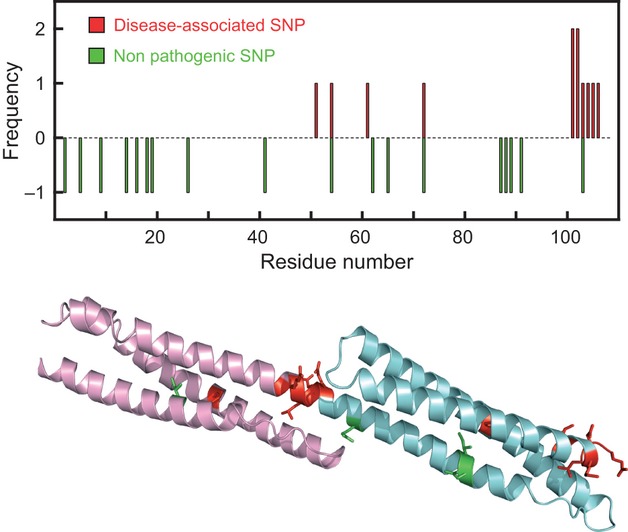
Location of nsSNPs in spectrin domains. Top: most disease-associated SNPs (red) are located in the region linking two domains, whereas the non-disease-associated SNPs (green) are distributed throughout the protein. Bottom: cartoon representation of the two-domain spectrin fragment R1617 (R16, pink; R17, blue), showing the locations of the residues mutated in this study (red and green).

To demonstrate this effect, we use the well-studied protein domains R15, R16 and R17 from chicken brain α-spectrin as model systems to study the effects of pathogenic mutations in human spectrin domains. These domains are a common component of proteins involved in cytoskeletal and membrane-associated structures, including spectrin, α-actinin and dystrophin 18, 19. Each spectrin repeat, or domain, is a stable, independently folding three-helix bundle comprising 106 amino acids. When arranged in tandem, a continuous α-helix links the C-terminus of one domain to the N-terminus of the following domain (see Fig. 1) 18–20. Although the interdomain interface is small (barely 800 Å^2^), there are significant interactions between adjacent domains 1,21,22.

Erythrocyte and brain spectrin most commonly exist as a tetramer: two antiparallel spectrin molecules, one α and one β, associate laterally to form heterodimers that further associate to form tetramers 23–25. Many disease-associated point mutations in erythrocyte spectrin have been mapped to these tetramerization sites, and may result in perturbation of the red blood cell structure, leading to haemolytic anaemias 26–28. However, over a dozen disease-causing mutations that are located distal to the tetramerization site have also been linked to haemolytic anaemias. Interestingly, many of these mutations occur at the spectrin repeat interface and many are mutations to proline 29. It has been suggested that some of these mutations may affect the cooperativity between spectrin domains 29. Using our model protein systems R15, R16 and R17, we take this analysis significantly further, specifically quantifying any changes in this ‘cooperativity’ upon mutation. We compare disease-related SNPs with others that are not associated with disease. Using a combination of thermodynamic and kinetic measurements, our results show that there is no clear pattern regarding the effect of each mutation on stability in the single-domain model proteins: the disease-causing mutations are only marginally more destabilizing than the non-disease-causing mutations. However, when the mutations are placed in the tandem spectrin models R1516 and R1617, a much clearer pattern emerges: our results suggest the disease-causing mutations disrupt the stabilizing interactions between adjacent domains, which results in a much larger decrease in stability than in the single-domain models. Our results also clearly show that in the tandem protein model, a mutation in one domain may have more of an effect on the stability of its neighbour than on itself: this behaviour is unlikely to be predicted by modelling programs. These findings highlight the importance of understanding the biophysical implications of a mutation in the context of neighbouring domains.

## Results

### Selecting mutations to study

Spectrin domains are 106-residue repeats, and the domain boundaries were as defined previously 21. We used UniProt (www.uniprot.org/uniprot) and the Human Genome Mutation Database (www.hgmd.cf.ac.uk) to compile a list of 12 disease-related and 20 non-disease-related nsSNPs in the spectrin domains of the human proteins α-and β-spectin (UniProt designations SPTA1_HUMAN and SPTB1_HUMAN), dystrophin (DMD_HUMAN) and α-actinin (ACTN3_HUMAN). These 32 SNPs were found in 24 spectrin domains spread between the four proteins. We ignored all mutations found at the tetramerization site in α-and β-spectrin. The sequences of these domains were compared with those of the previously well-characterized chicken brain α-spectrin R15, R16 and R17, using alignments compiled using ClustalW (www.ebi.ac.uk/tools/msa/clustalw2). These were subsequently verified by comparison with the Pfam alignment (www.pfam.sanger.ac.uk) (Fig. S1). As observed previously, most of the disease-related mutations are found at the domain boundaries, while the non-disease-related sequence changes are found throughout the protein (Fig. 1). We identified sites in our model spectrin domains at which we could create a point mutation that was analogous to the amino acid change found in the host domain (Fig. 1 and Table 1). In some cases, we created a mutation that was an exact match to the disease-related mutation (e.g. L104P as a model for L260P, in all three model proteins); in others, we matched residue type (e.g. I51P in R15 to model L207P). This resulted in seven disease-related mutations and four non-disease-associated mutations. Some of these mutations were created in all three model protein systems and others in only one. In total, we characterized 24 single-domain mutant model proteins (Table 1 and Fig. S2).

**Table 1:** Characterization of single-domain mutant proteins. Note that our spectrin domains have extensions at either end 38. Residue 1 of the R16 106-residue spectrin repeat was thus numbered residue 5 in our previous work.

	Comment	Model protein	Mutation created in model	Helix, position, exposure^a^	Change in free energy of unfolding on mutation (ΔΔ*G*_D−N_, kcal.mol^−1^)
Disease-associated SNPs					
G151D (α-spectrin)	Replacement of Gly by charged residue	R16	G101D	C, end, buried	1.3 ± 0.3
L207P (α-spectrin)	Replacement of hydrophobic residue by Pro	R15	I51P	B, mid, buried	Unfolded (> 6.4)
		R16	L51P		Insoluble (> 6.1)
		R17	F51P		1.3 ± 0.4
L260P (α-spectrin)	Replacement of hydrophobic residue by Pro	R15	L104P	C, end, buried	2.3 ± 0.4
		R16	L104P		1.5 ± 0.4
		R17	L104P		0.7 ± 0.3
S261P (α-spectrin)	Replacement of polar residue by Pro	R15	N105P	C, end, surface	1.3 ± 0.4
		R16	E105P		1.2 ± 0.4
		R17	D105P		2.4 ± 0.3
Q471P (α-spectrin)	Replacement of polar residue by Pro	R15	K103P	C, end, surface	1.4 ± 0.4
		R16	R103P		2.0 ± 0.3
		R17	K103P		3.6 ± 0.3
H469P (α-spectrin)	Replacement of polar residue by Pro	R15	R101P	C, end, surface	3.2 ± 0.3
		R16	G101P		1.4 ± 0.3
		R17	K101P		4.1 ± 0.4
D791E (α-spectrin)	Replacement of acidic residue by another acidic residue	R15	E106D	C, end, surface	0.0 ± 0.3
		R16	E106D		0.3 ± 0.3
		R17	E106D		0.6 ± 0.4
Non-disease-associated SNPs					
I809V (α-spectrin)	Replacement of hydrophobic residue by smaller hydrophobic	R16	I18V^b^	A, mid, buried	1.4 ± 0.3
		R17	I18V^c^		1.3 ± 0.4
N438S (β-spectrin)	Replacement of polar residue by smaller polar	R17	N19S	A, mid, surface	0.3 ± 0.3
H1373R (β-spectrin)	Replacement of polar residue by charged	R16	R103H	C, end, surface	0.4 ± 0.4
Q2937R (dystrophin)	Replacement of polar residue by charged	R17	Q5R	A, start, surface	0.5 ± 0.3

a Buried residues have a side chain with a solvent-accessible surface area < 10%.

b Data taken from 41.

c Data taken from 42.

### There is no clear difference in the effects of disease and non-disease mutations on the stability of isolated domains

The effect of mutation on the stability of the single-domain model proteins was determined using the equilibrium denaturation method (Table 1 and Fig. S3). Interestingly, equivalent mutations did not always have the same effect on the various model proteins, in contrast to previous observations on Ig-like domains 14. This may reflect the greater structural plasticity of the helix-bundle proteins when compared to the β-sandwich Greek key Ig-like domains. In Ig-like domains, there is significant conservation of core residues [Bibr b30],[Bibr b31], whereas only two of the 106 residues in spectrin domains are conserved to any significant extent: Trp 17 and Leu 104. Most importantly, and perhaps surprisingly, many of the mutations that result in disease were not strongly destabilizing, and there was certainly no clear distinction between the pathogenic and non-pathogenic datasets. Only one mutation, L207P, which involves substitution of a buried hydrophobic residue by a proline residue in the middle of helix B, caused a significant loss of stability in our model proteins, resulting in unstable R15 and R16 domains. Interestingly, even this change is inconsistent, as when there is a larger hydrophobic residue (Phe) at this position (in R17), insertion of the helix-breaking Pro residue is tolerated, possibly reflecting the plasticity of these domains, i.e. if there is a large enough cavity, the protein may accommodate Pro even in the centre of the helix.

### Determining the effects of mutation in multidomain proteins

Most of the pathogenic mutations are clustered in the linker helix between domains. It has previously been demonstrated that spectrin domains are stabilized by their neighbours 21,22. These stabilizing interactions are dependent on the contiguous helix between the domains (Fig. 1). Thus, to mimic both pathogenic and non-pathogenic SNPs, we created a number of mutations in the model two-domain proteins R1516 and R1617, which have the same structure and the same linking helix (Table 2 and Fig. S4). We have previously shown that it is not possible to determine the stability of a two-domain protein by simple equilibrium measurements: it is necessary to determine the folding and unfolding rate constants of each domain (*k*_f_ and *k*_u_, respectively), both alone and in the two-domain system, to determine the effect of a mutation on the stability of a two-domain protein 32–34. Thus we performed a series of kinetic experiments. We determined *k*_f_ and *k*_u_, extrapolated to 0 m denaturant, for each domain in the two-domain protein constructs. The method of analysis is explained in detail in Doc. S1 and Figs S5–S10, which include some sample kinetic chevron plots. The results are given in Table 2. Note that the domain with the mutation is marked with an asterisk; thus, for instance, R1617*I18V has an I→V substitution at position 18 in R17.

**Table 2:** Characterization of two-domain mutant proteins

1	2	3	4	5	6	7	8	9	10	11	12	13	14
	Kinetics								Thermodynamics				
	R16 isolated domain		R16 in R1617^a^		R17 isolated domain		R17 in R1617^b^		Loss of stability of R16 domain (kcal·mol^−1^)^c^		Loss of stability of R17 domain (kcal·mol^−1^)^c^		Total loss in stability of R1617 on mutation (kcal·mol^−1^)^c^
	*k*_f_ (s^−1^)	*k*_u_ (s^−1^)	*k*_f_ (s^−1^)	*k*_u_ (s^−1^)	*k*_f_ (s^−1^)	*k*_u_ (s^−1^)	*k*_f_ (s^−1^)	*k*_u_ (s^−1^)	In R16 alone	In R1617	In R17 alone	In R1617	
WT	130	0.0032	140	0.00090	27	0.00071	860	0.00012					
Mutations in R16													
***I18V***	***66***	***0.019***	***52***	***0.0053***	***NA***	***NA***	***980***	***0.00021***	***1.4***	***1.6***	***NA***	***0.2***	***1.8***
G101D	110	0.047	110	0.058	NA	NA	20	0.00032	1.7	2.6	NA	2.8	5.4
G101P	100	0.10	100	0.050	NA	NA	20	0.00036	2.2	2.6	NA	2.9	5.4
R103P	110	0.070	97	0.020	NA	NA	17	0.00034	1.9	2.0	NA	2.9	5.0
***R103H***	***110***	***0.0064***	***150***	***0.0012***	***NA***	***NA***	***1300***	***0.00016***	***0.5***	***0.1***	***NA***	***−0.1***	***0.0***
L104P	100	0.072	96	0.053	NA	NA	14	0.00041	2.0	2.6	NA	3.1	5.7
E105P	97	0.033	160	0.019	NA	NA	13	0.00043	1.5	1.7	NA	3.2	5.0
E106D	130	0.0053	110	0.00081	NA	NA	1000	0.000075	0.3	0	NA	−0.5	−0.4
Mutations in R17													
***Q5R***	***NA***	***NA***	***110***	***0.0013***	***18***	***0.00089***	***460***	***0.00019***	***NA***	***0.3***	***0.4***	***0.6***	***1.0***
***I18V***	***NA***	***NA***	***130***	***0.00077***	***7.6***	***0.0026***	***560***	***0.0011***	***NA***	***−0.1***	***1.5***	***1.6***	***1.5***
***N19S***	***NA***	***NA***	***120***	***0.0010***	***9.0***	***0.00080***	***810***	***0.00018***	***NA***	***0.1***	***0.7***	***0.3***	***0.4***

	R15 isolated domain		R15 in R1516^d^		R16 isolated domain		R16 in R1516^e^		Loss of stability of R15 domain (kcal·mol^−1^)^c^		Loss of stability of R16 domain (kcal·mol^−1^)^c^		Total loss in stability of R1516 on mutation (kcal·mol^−1^)^c^
	*k*_f_ (s^−1^)	*k*_u_ (s^−1^)	*k*_f_ (s^−1^)	*k*_u_ (s^−1^)	*k*_f_ (s^−1^)	*k*_u_ (s^−1^)	*k*_f_ (s^−1^)	*k*_u_ (s^−1^)	In R15 alone	In R1516	In R16 alone	In R1516	
WT	28 000	2.1	20 000	0.071	130	0.0032	730	0.00074					
Mutations in R15													
E106D	26 000	2.9	17 000	0.21	NA	NA	840	0.00092	0.2	0.7	NA	0.0	0.8
N105P	23 000	11	13 000	15	NA	NA	87	0.0033	1.1	3.4	NA	2.1	5.6

*k*_f_ and *k*_u_ are the rate constants for folding and unfolding, respectively, extrapolated to 0 m denaturant. The non-disease-related mutations are shown in bold and italics. The mutants I18V in R17 and N105P in R15 are discussed in detail in the text and are highlighted in red and blue, respectively.

a As R16 folds first and unfolds last, the R16 kinetic parameters are always determined in the presence of an unfolded R17 neighbour.

b As R16 folds first and unfolds last, the R17 kinetic parameters are always determined in the presence of a folded R16 neighbour.

c The stability changes are calculated using the kinetic data presented, using the relationship Δ*G*_D-N_ = −*RT* ln(*k*_u_/*k*_f_). For the single domains, all values are very close to those determined by equilibrium measurements (as reported in Table 1), except for G101P. The kinetic measurements are more subject to experimental error because of the extrapolation of the unfolding data to 0 m denaturant. The errors for these estimates of Δ*G*_D-N_ are generally in the order of 0.1–0.3 kcal·mol^−1^.

d As R15 folds first and unfolds last, the R15 kinetic parameters are always determined in the presence of an unfolded R16 neighbour.

e As R15 folds first and unfolds last, the R16 kinetic parameters are always determined in the presence of a folded R15 neighbour.

For the non-pathogenic mutations, we found that the stabilizing interactions between the domains were retained, such that the total loss of stability in the two-domain protein was essentially the same as the loss of stability in the single domain. As an example, the mutation I18V in R17 (Table 2, highlighted in red) destabilizes single-domain R17 by ∼ 1.5 kcal·mol^−1^ (Table 2, column 12) and the R17 domain in R1617 by the same amount (1.6 kcal·mol^−1^) (Table 2, column 13). In both cases, the destabilization arises from slowing the rate of folding and increasing the rate of unfolding. R17 is stabilized by wild-type (WT) R16 in R1617 mainly by speeding the folding by ∼ 30-fold. In R1617*I18V, the R17 domain still folds very rapidly *k*_f_ of ∼ 560 s^−1^ (column 8) compared with the mutant single-domain protein *k*_f_ of ∼ 7.6 s^−1^ (column 6), and the stability of the R16 domain in R1617*I18V is the same as that in WT R1617 (column 11). Thus the stabilizing interactions are retained and the loss of stability of the two-domain protein is the same as the loss of stability of the single-domain protein (columns 12 and 14). We found essentially the same results for all the non-pathogenic mutations. Thus the overall loss of stability resulting from these non-pathogenic mutations, even in the multidomain context, was < 2.0 kcal·mol^−1^ in all cases. This is consistent with the threshold that has been observed previously for other proteins 9,14,14–16.

**Fig 2:**
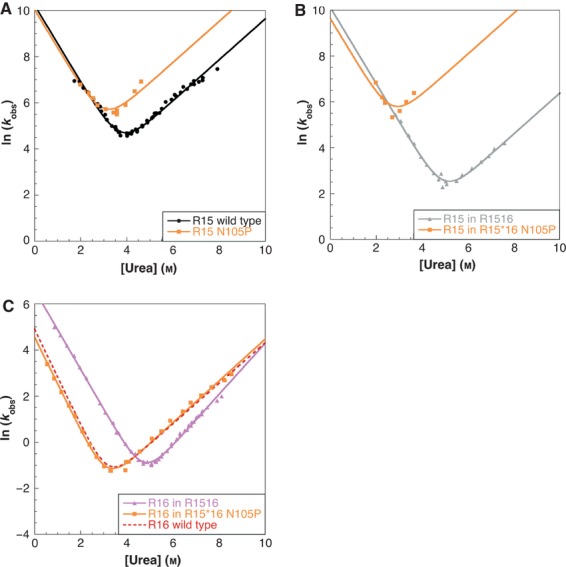
The mutation N105P in R15 results in loss of stabilizing interactions between the domains in R1516. (A) Single domains: WT R15 (black) and R15 N105P (orange). The folding rate is essentially unaffected by the mutation; however, the mutation causes an increase in the unfolding rate of R15. (B) R15 in WT R1516 (grey) and mutant R1516 (orange). The mutation affects the R15 domain exactly as in the single-domain protein. The unfolding rate of the mutant is now significantly faster than WT R15 in R1516. (C) R16 in WT R1516 (pink) and mutant R1516 (orange). The effect is dramatic. The mutant protein folds much more slowly and unfolds faster than WT. In fact, it folds just like the WT R16 single-domain protein. Thus folding of R16 in the mutant R1516 is essentially identical to the WT form (included for comparison, red dotted line). All the stabilizing interactions between R15 and R16 have been lost.

However, we obtained very different results for the pathogenic mutations: for most pathogenic mutations, the loss of stability comprises the loss of stability of the parent domain plus the loss of all the stabilizing reactions between the domains. As an example, the pathogenic-like mutation N105P in R15 and R1516 destabilizes single-domain R15 by ∼ 1.1 kcal·mol^−1^: although the mutant folds at approximately the same speed as WT R15, it unfolds more rapidly (the rate constant for unfolding, *k*_u_, is approximately five times larger than WT) (Fig. 2A). However, the same mutation in R1516 has a much greater effect on the R15 domain. The kinetic data for this mutation are shown in Fig. 2 and Table 2 (highlighted in blue). Figure 2B shows that, although the mutant R15 domain still folds as fast as WT R15 in R1516, it now unfolds much more rapidly than WT, i.e. *k*_u_ is increased 200-fold, from 0.071 s^−1^ (WT) to 15 s^−1^ (mutant). This means that the mutation N105P destabilizes R15 in R1516 by ∼ 3.4 kcal·mol^−1^. Moreover, R16, which was originally stabilized by R15 (folding more rapidly and unfolding more slowly), loses this stability (Fig. 2C). R16 in R15*16 N105P now behaves as if it were a single domain, with a loss of stability of ∼ 2 kcal·mol^−1^. Thus, as seen in Table 2, column 14, the mutation N109P causes a total destabilisation of 5.6 kcal·mol^−1^, rather than the loss of 1.2 kcal·mol^−1^ found for the mutation in isolated R15.

## Discussion

### Studying tandem spectrin domains: use of model protein systems

**Fig 3:**
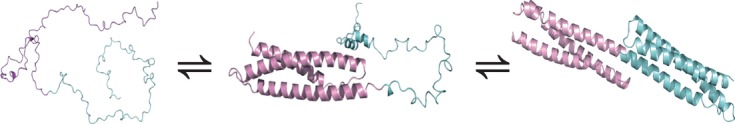
The folding pathways of R1516 and R1617 are essentially the same. The N-terminal domain (pink) folds first, followed by the C-terminal domain (blue). Unfolding is the reverse of this process. This is a consequence of the relative folding and unfolding rate constants (Table 2).

Early equilibrium studies showed that spectrin domains were stabilized by their neighbours 21,22. This ‘cooperativity’ was ascribed to the linking helix region. However, this effect could not be effectively quantified until kinetic experiments were introduced 32,34. Such kinetic studies may be very difficult to undertake. The domains must be investigated both in isolation and in tandem, and the kinetics may be extraordinarily difficult to disentangle 34. The results of these studies were in some respects quite surprising. Using spectrin repeats R15, R16 and R17 and tandem domains R1516, R1617 and R151617, with other extended constructs, we were able to show that R15 and R16 are both stabilized by a simple extension at the C-terminus (but not at the N-terminus) 35. In other words, R15 is stabilized even by unfolded R16, and R16 is stabilized even by unfolded R17, in both cases by ∼ 1–2 kcal·mol^−1^. However, there is also a mutual stabilization between neighbouring domains when both domains are folded (by 2–3 kcal·mol^−1^ in both cases). The folding pathways for R1516 and R1617 are essentially the same (Fig. 3) 32,35. First the N-terminal domain folds, then the C-terminal domain. Thus R15 folds before R16 in R1516, and R16 folds before R17 in R1617. The order of unfolding is the reverse, first the C-terminal domain unfolds (R16 in R1516 and R17 in R1617), and then the N-terminal domain unfolds. The consequence of this is that, in kinetic experiments, we may investigate the folding behaviour of the N-terminal domain in the presence of an unfolded C-terminal domain (but not in the presence of a folded one), and we may investigate the folding behaviour of the C-terminal domain in the presence of a folded N-terminal domain (but not in the presence of an unfolded one). However, this is enough to enable us to determine the stability of the entire system, because (in these two-state proteins), the free energy of unfolding (Δ*G*) may be calculated from the folding and unfolding rate constants Δ*G* = −*RT* ln(*k*_u_/*k*_f_). The stability of the entire two-domain spectrin construct is thus:

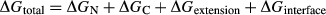

where Δ*G*_N_ and Δ*G*_C_ are the free energies of unfolding of the N-and C-terminal domains, respectively, Δ*G*_extension_ is the gain in free energy of the N-terminal domain from simple extension (by the unfolded C-terminal domain), and Δ*G*_interface_ is the stabilization of one domain by its folded neighbour.

As a full analysis of the folding of individual domains and the sometimes very complex two-domain constructs is necessary to fully characterize spectrin repeats, a systematic analysis of the thermodynamic and kinetic effects of pathogenic (and non-pathogenic) variants in their natural environment is simply prohibitive. Here, for instance, we investigate nine mutations in seven different α-spectrin domains, as well as two mutations in different domains of β-spectrin and one mutation of dystrophin. We had previously characterized three different wild-type single repeat proteins plus two tandems 32,37. To have achieved these results using the natural spectrin domains, we would have had to characterize at least ten new single wild-type domain constructs and at least nine wild-type two-domain constructs. The use of model proteins that have been previously well characterized is a useful alternative strategy, in particular if the same or a closely equivalent mutation may be created in more than one model domain.

### Comparing the effects of mutations in single-and multidomain systems

Here we find that the point mutations in the single domains result in proteins that are somewhat destabilized relative to the WT domain (Table 1). This is manifested by slower folding and faster unfolding. However, none of the mutations, with the exception of those that mimic L207P, are exceptionally destabilizing. Indeed, other L→P substitutions (e.g. those that mimic L260P) are benign at the single-domain level. Certainly, the difference between pathogenic and non-pathogenic variants is not clear from the isolated domains.

**Fig 4:**
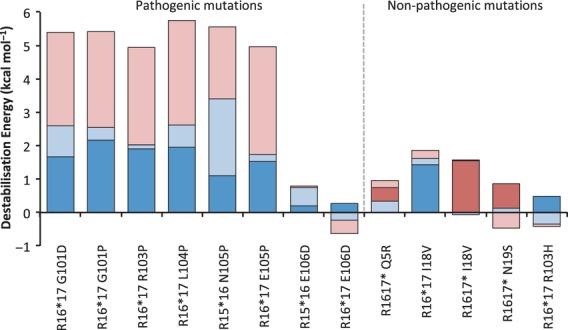
Pathogenic mutations are far more destabilizing than non-pathogenic mutations in the natural tandem repeat context. Thirteen mutations were created in both single-and multidomain contexts. Where the multidomain protein is R1516, ‘domain 1’ is R15; where the protein is R1617, ‘domain 1’ indicates R16. The ‘expected destabilization’ (domain 1, dark blue; domain 2, red) is the effect of the mutation on the single-domain protein. The ‘extra destabilization’ (domain 1, light blue; domain 2, pink) is the extra destabilization observed in the multidomain protein. Apart from E106D, all the pathogenic mutations result in loss of inter-domain stabilizing interactions. This results in an overall destabilization of > 5 kcal·mol^−1^. The non-pathogenic mutations all maintain the inter-domain interactions, and the loss in stability is essentially that ‘expected’ from the single-domain protein results, i.e. below 2 kcal·mol^−1^.

For the non-pathogenic mutations, the effect of a mutation in the two-domain system is approximately the same as the effect in the isolated domain. All stabilizing interactions between the domains are intact. This is shown in Fig. 4. A mutation in one domain has no effect on the stability of the neighbouring domain.

However, the case for the pathogenic mutations is entirely different. With one exception, all pathogenic mutations caused a loss of the stabilizing interactions between the domains. The result is quite remarkable (Fig. 4). Thus, the pathogenic mutations, again with one exception, result in a loss of stability of the system of ∼ 5 kcal·mol^−1^ or more, an increase of ∼ 3.5–4 kcal·mol^−1^ over and above the effect of the mutation in the single domain. The one exception was the mutation E106D in both R15*16 and R16*17. This mutation was created to model the mutation D791E in human α-spectrin, which has been shown to cause hereditary elliptocytosis 36. This substitution has very little effect in any of the single domains, and no effect at all on the inter-domain stabilizing interactions. We infer that this mutation has site-specific effects within the spectrin molecule, possibly removing an unknown interaction site within the human spectrin heterodimer or an interaction with other cytoskeletal proteins.

Undoubtedly, spectrin repeats gain significant stability through nearest-neighbour effects, mediated through the linker helix. The stabilization conferred by these effects (∼ 4 kcal·mol^−1^ per interface) is very significant when compared to the stability of spectrin domains that we have studied, which ranges from 3.5–6.5 kcal·mol^−1^. In a previous study, Johnson *et al*. 29 investigated a single pathogenic mutation (Q471P) in the context of a five-repeat fragment of α-spectrin. The mutant protein had a lower thermal stability relative to WT, consistent with the experiments reported here. However, a detailed thermodynamic study was not performed, and indeed is not possible using purely equilibrium methods 33. Here we have used model protein systems to help illustrate the effects of pathogenic mutations that destroy the stabilizing interactions between domains. What is quite remarkable is the much higher destabilisation of the mutations that result in disease compared to non-pathogenic mutations in the same model systems (Fig. 4). We have also identified a mutation with a functional effect, perhaps disruption of a binding site, (D791E), as well as a mutation that is likely to cause disease by directly destabilizing a single domain (L207P), although, in the latter case, we note that, if a domain is destabilized to the extent that it is unfolded, inter-domain interactions will be lost. Importantly, structural modelling in the absence of biophysical data would not have predicted such drastic effects for the mutations investigated here.

## Experimental procedures

### Protein expression and purification

The mutants were selected according to sequence alignments (Figs S1 and S2). Mutagenesis was performed using a QuikChange site-directed mutagenesis kit (Agilent Technologies, Santa Clara, CA, USA), and the identity of the mutants was confirmed by DNA sequencing. Protein expression and purification were performed as described previously 37. Note that, in our previous studies, we always used extended domains to ensure that we did not artificially destabilize the proteins by making domain boundaries too short 37–39. We use the same extended domains here, but number them to agree with the numbering convention described by MacDonald and Pozharski 21. Thus, the numbering in the present paper is different from that in our previous work. For example, the residue numbered 1 in this paper is in fact the 5th residue in our previous studies 40–42.

### Stability measurements

The stability of the mutant single-domain proteins was determined by equilibrium denaturation using urea as the denaturant. Folding was monitored on the basis of intrinsic tryptophan fluorescence, measured using a Perkin Elmer (Waltham, MA, USA) fluorescence spectrometer with a final protein concentration of 1 μm. Dithiothreitol was added to a final concentration of 5 mm for R17 and R1617 proteins. All experiments were performed at 25 ± 0.1 °C in 50 mm sodium phosphate buffer (pH 7.0). The data were fitted to a two-state transition curve as described previously 43,44.

### Kinetic measurements

Kinetic experiments on the mutant proteins were performed using a stopped-flow fluorimeter (Applied Photophysics Leatherhead, Surrey, UK SX.18MX) at 25 ± 0.1 °C in 50 mm sodium phosphate buffer (pH 7.0). The final protein concentration was 1 μm, with 5 mm dithiothreitol added for R17 and R1617. Samples were excited at a wavelength of 280 nm, and the emission was monitored above 320 nm. At least six overlying traces were obtained at all concentrations of urea. Single-jump experiments on all proteins were performed using 10: 1 mixing (buffer:protein). Double-jump experiments were performed on tandem repeats R1516 and R1617. For R1617, interrupted unfolding experiments allowed the folding rate of R17 to be observed: proteins were initially unfolded in urea at a 1:5 ratio (protein:urea) for a delay time of 500 ms, and then jumped into refolding solutions at 1:10 ratio (protein:buffer). For R1516, interrupted refolding experiments allowed the unfolding rate of R15 to be observed: unfolded protein in 8 m urea was allowed to refold to a final concentration of 4 m with a delay time of 100 ms, and then jumped into unfolding solutions. Data for all experiments were fitted using Kaleidagraph (Synergy Software, Reading, PA, USA).

## Abbreviations

Δ*G*: free energy of unfolding
Ig-like: immunoglobulin-like
*k*_f_ and *k*_u_: refolding and unfolding rate constants, respectively, in water
ns: non-synonymous
SNP: single-nucleotide polymorphism

## Notes

**Doc S1** Analysis of kinetic data for two-domain spectrin fragments. **Fig S1** Alignments used to identify suitable mutation sites for this study. **Fig S2** Model proteins used in this study. **Fig S3** Equilibrium denaturation curves for all single-domain proteins described in this study. **Fig S4** The linking helix is conserved in spectrin repeats. **Fig S5** Chevron plot showing folding and unfolding arms. **Fig S6** Comparison of the folding of R15 and R16 alone and in R1516. **Fig S7** Comparison of the folding of R16 and R17 alone and in R1617. **Fig S8** The mutation E106D does not disrupt inter-domain interactions in R1516. **Fig S9** The mutation I18V does not disrupt inter-domain interactions in R1617. **Fig S10** The mutation E105P destroys interactions between the domains.
