# Supplementary material for: Understanding pathogenic single-nucleotide polymorphisms in multidomain proteins – studies of isolated domains are not enough
Source: FEBS J. 2013 Jan 16;280(4):1018–27. doi: 10.1111/febs.12094 (PMC3790955; doi:10.1111/febs.12094)
Supplement: Supplementary file 1 — Doc S1 Analysis of kinetic data for two-domain spectrin fragments. Fig S1 Alignments used to identify suitable mutation sites for this study. Fig S2 Model proteins used in this study. Fig S3 Equilibrium denaturation curves for all single-domain proteins described in this study. Fig S4 The linking helix is conserved in spectrin repeats. Fig S5 Chevron plot showing folding and unfolding arms. Fig S6 Comparison of the folding of R15 and R16 alone and in R1516. Fig S7 Comparison of the folding of R16 and R17 alone and in R1617. Fig S8 The mutation E106D does not disrupt inter-domain interactions in R1516. Fig S9 The mutation I18V does not disrupt inter-domain interactions in R1617. Fig S10 The mutation E105P destroys interactions between the domains. [file febs0280-1018-sd1.zip › febs12094-sup-0001-FigS1-S10.pdf]

the FEBS Journal

www.febsjournal.org

Understanding pathogenic single-nucleotide polymorphisms in multidomain proteins – studies of isolated domains are not enough

Lucy G. Randles, Gwen J. S. Dawes, Beth G. Wensley, Annette Steward, Adrian A. Nickson1 and Jane Clarke

DOI: 10.1111/febs.12094

Supplementary Information for:

Understanding pathogenic Single Nucleotide Polymorphisms in multidomain proteins - studies of isolated domains are not enough

Lucy Randles, Gwen Dawes, Beth Wensley, Annette Steward, Adrian Nickson and Jane Clarke

This Supplementary File has 4 Supplementary figures, (S1-S4), as referenced in the main body of the text.

It also contains a document (Doc. S1) and six additional figures, (S5 – S10), that describe the analysis of the kinetic data to a non-specialist audience.

Data on the individual wild-type proteins (R15, R16 and R17) are taken from a paper by Scott *et al.*, [1], whereas all data on the tandem repeat wild-type proteins (R1516 and R1617) was first reported in a paper by Batey *et al.* [2].

1. Scott, K. A., Batey, S., Hooton, K. A. & Clarke, J. (2004) The folding of spectrin domains I: wild-type domains have the same stability but very different kinetic properties, *J Mol Biol.* **344**, 195-205.
2. Batey, S. & Clarke, J. (2006) Apparent cooperativity in the folding of multidomain proteins depends on the relative rates of folding of the constituent domains, *Proc Natl Acad Sci USA.* **103**, 18113-18118.

Doc. S1. Analysis of kinetic data for two-domain spectrin fragments

(1) The chevron plot

The logarithm of the rate constants for folding and unfolding (k_f and k_u, respectively) are displayed on a so-called chevron plot, shown in Figure S5. The "folding arm" on the left hand side of the plot shows that folding gets slower as the concentration of denaturant increases (the slope of the chevron plot is negative). The "unfolding arm" on the right hand side reflects the fact that unfolding gets faster as more denaturant is added. From extrapolation of the folding and the unfolding arms it is possible to determine k_f and k_u at 0 M denaturant and thus determine the stability (free energy of unfolding, $\Delta G_{D\text{-}N} = RT\ln(k_f/k_u)$) of the protein if the folding is 2-state.

(2) Comparing the folding of spectrin domains in the 2-domain proteins R1516 and R1617

In these 2-domain proteins the N-terminal domain folds first, followed by the C-terminal domain. In both, the C-terminal domain unfolds first, followed by the N-terminal domain (See Figure 3 main text). Thus, in R1516, R15 is folding and unfolding while attached to an unfolded R16 domain, and R16 is folding and unfolding while attached to a folded R15 domain. Likewise in R1617, R16 is folding and unfolding while attached to an unfolded R17 domain, and R17 is folding and unfolding while attached to a folded R16 domain. In Figure S6, below, we show the folding of R1516.

In Figure S6A we see the R15 domain alone (black) and in R1516 (grey). R15 folds at the same rate in R1516 as it does on its own, but unfolding is slowed significantly (about 65-fold). Thus we deduce that R15 is stabilized ~ 2 kcal mol^{-1} by *unfolded* R16. In Figure S6B we see R16 alone (red) and in R1516 (magenta). R16 folds faster (about 6-fold) and unfolds more slowly (about 3.5-fold) in the presence of R15. Thus we deduce that R16 is stabilized by ~ 1.7 kcal mol^{-1} by *folded* R15.

In Figure S7 we show the folding of R1617, which is more complex. There is "roll-over" in the folding arm. This is because R1617 forms a partially structured folding intermediate that is not seen in either domain alone. However, we can analyze the data to determine the folding rate of the R16 and R17 domains directly from the denatured state, which is what is measured in the single domains (fitted magenta line).

In Figure S7A we see R16 alone (red) and in R1617 (magenta, with the fitting shown by a solid line). There is no change in the rate constants for folding, but unfolding is slowed, by about 3-fold. Thus we deduce that R16 is stabilized ~ 0.6 kcal mol^{-1} by *unfolded* R17. In Figure S7B we show R17 alone (dark blue) and in R1617 (light blue). R17 both folds faster (~ 30-fold) and unfolds more slowly (~6-fold) in R1617. R17 is stabilized about 4 kcal mol^{-1} by *folded* R16.

(3) Analysis of mutant proteins

In the main text we have shown the chevron plots for R15*16 N105P. Here we describe in detail how to read three other chevron plots: first, R15*16 E106D, where there is no disruption to the interaction between R15 and R16 in R1516, (Figure S8 A-C); second, R16*17 I18V, where there is no disruption to the interaction between R16 and R17 in R1617, (Figure S9 A-C); finally, R16*R17 E105P, a mutation that disrupts the interactions between R16 and R17 in the 2-domain protein, (Figure S10 A-C).

```
                                Helix A                        Helix B
                  hhhhhhhhhhhhhhhhhhhhhhhhhhh        hhhhhhhhhhhhhhhh

R15_chick       ...NKQQNFNTGIKDFDFWLSE..VEALLASEDYGKDLASVNNLLKKHQLLEADI
R16_chick       ..SHRLHQFFRDMDDEESWIKE..KKLLVSSEDYGRDLTGVQNLRKKHKRLEAEL
R17_chick       ..SLEYQQFVANVEEEEAWINE..KMTLVASEDYGDTLAAIQGLLKKHEAFETDF
α_human (1)     ...YHLQVFKRDADDLGKWIME...KVNILTDKSY.EDPTNIQGKYQKHQSLEAEV
α_human (2)     ...LKFQQYVQECADILEWIGD..KEAIATSVELGEDWERTEVLHKKFEDFQVEL
α_human (4)     ...YWYHRFSSDFDELSGWMNEK..TAAINADELPTDVAGGEVLLDRHQQHKHEI
α_human (7)     ...NQQLQFENNAEDLQRWLED..VEWQVTSEDYGKGLAEVQNRLRKHGLLESAV
α_human (8)     ...LHLQLICRDTEDEEAWIQE..TEPSATSTYLGKDLIASKKLLNRHRVILENI
α_human (9)     ...VQFQQYLADLHEAETWIRE..KEPIVDNTNYGADEEAAGALLKKHEAFLLDL
β_human (x)     ...QLARRFDRKAAMRETWLNEN.QRLVAQDNFGYDLAAVEAAKKKHEAIETDT
β_human (x)     ...SQLQAFLQDLDDFQAWLSIT..QKAVASEDMPESLPEAEQLLQQHAGIKDEI
β_human (x)     ...LGFQEFQKDAKQAEAILSN..QEYTLAHLEPPDSLEAAEAGIRKFEDFLGSM
β_human (x)     ...LELQNFLQNCQELTLWIND...KLLTSQDVSYDEARNLHNKWLKHQAFVAEL
β_human (x)     ....SDLRLQT.HADLNKWISA..MEDQLRSDDPGKDLTSVNRMLAKLKRVEDQV
β_human (x)     ...NEAQQYYLDADEAEAWIGE..QELYVISDEIPKDEEGAIVMLKRHLRQQRAV
DMD_human (x)   ...VNLDRYQTALEEVLSWLLS.AEDTLQAQGEISNDVEVVKDQFHTHEGYMMDL
DMD_human (x)   ...EIRKRLDVDITELHSWITRSEAVLQSPEFAIFRKEGNFSDLKEKVNAIEREK
DMD_human (x)   ...NNIIAFYNQLQQLEQMTTT..AENWLKIQPTTPSEPTAIKSQLKICKDEVNR
DMD_human (x)   ...EKTVSLQKDLSEMHEWMTQA.EEEYLERDFEYKTPDELQKAVEEMKRAKEEA
DMD_human (x)   ...ACWHELLSYLEKANKWLNEV.EFKLKTTENIPGGAEEISEVLDSLENL
DMD_human (x)   RLFQKPANFEQRLQESKMILDEV..KMHLPALETKSVEQEVVQSQLNHCVNLYKS
DMD_human (x)   ...KLSRKMRKEMNVLTEWLAAT.DMELTKRSAVEGMPSNLDSEVAWGKATQKEI
DMD_human (x)   ...KHMETFDQNVDHITKWIIQ...ADTLLDESEKKKPQQKEDVLKRLKAELNDI
DMD_human (x)   ...EKWRRFHYDIKIFNQWLTE...AEQFLRKTQIPENWEHAKYKWYLKELQDGI
DMD_human (x)   ...NILSEFQRDLNEFVLWLEEA..DNIASIPLEPGKEQQLKEKLEQVKLLVEEL
DMD_human (x)   ...ERLQELQEATDELDLKLRQAEVIKGSWQPVGDLLIDSLQDHLEKVKALRGEI
ACTN3-human     ...QLQLEFARRAAPFNNWLDG..AVEDLQDVWLVHSVEETQSLLTAHDQFKATL

                        Helix B                           Helix C
                hhhhhhhhhhhhhhhhhhhhhh    hhhhhhhhhhhhhhhhhhhhhhhhhhhhhhhhhhhhhhhh

R15_chick       SAH.EDRLKDLNSQADSLMTSSAFDTSQVKDKR.......ETINGRFQRIKSMAAARRAKLNE
R16_chick       AAH.EPAIQGVLDTGKKLSDDNTIGKEEIQQRL.......AQFVDHWKELKQLAAARGQRLEE
R17_chick       TVH.KDRVNDVCANGEDLIKKNNHHVENITAKM.......KGLKGKVSDLEKAAAQRKAKLDENSAFLQ
α_human (1)     QTK.SRLMSELEKTREERFTMGHSAHEETKAHI.......EELRHLWDLLLELTLEKGDQLLR
α_human (2)     VAK.EGRVVEVNQYANECAEENHPDLPLIQSKQ.......NEVNAAWERLRGLALQRQKALSN
α_human (4)     DSY.DDRFQSADETGQDLVNANHEASDEVREKM.......EILDNNWTALLELWDERHRQYEQ
α_human (7)     AAR.QDQVDILTDLAAYFEEIGHPDSKDIRARQ.......ESLVCRFEALKEPLATRKKKLLDL
α_human (8)     ASH.EPRIQEITERGNKMVEEGHFAAEDVASRV.......KSLNQNMESLRARAARRQNDLEA
α_human (9)     NSF.GDSMKALRNQANACQQQQAAPVEGVAGE............QRVMALYDFQARSPREVTM
β_human (x)     AAY.EERVRALEDLAQELEKENYHDQKRITARK.......DNILRLWSYLQELLQSRRQRLET
β_human (x)     DGH.QDSYQRVKESGEKVIQGQTDPEYLLLGQR......LEGLDTGWDALGRMWESRSHTLA
β_human (x)     ENN.RDKVLSPVDSGNKLVAEGNLYSDKIKEKV.......QLIEDRHRKNNEKAQEASVLLRD
β_human (x)     ASH.EGWLENIDAEGKQLMDEKPQFTALVSQKL.......EALHRLWDELQATTKEKTQHLSA
β_human (x)     NVR.KEELGELFAQVPSMGEEGGDADLSIEKR..............FLDLLEPLGRRKKQLES
β_human (x)     EDY.GRNIKQLASRAQGLLSAGHPEGEQIIRLQG.......QVDKHYAGLKDVAEERKRKLEN
DMD_human (x)   TAH.QGRVGNILQLGSKLIGTGKLSEDEETEVQEQMN....LLNSRWECLRVASMEKQSNLHR
DMD_human (x)   AEK.FRKLQDASRSAQALVEQMVNEGVNADSIKQAS....EQLNSRWIEFCQLLSERLNWLEY
DMD_human (x)   LSGLQPQIERLKIQSIALKEKGQGPMFLDADFV........AFTNHFKQVFSDVQAREKELQT
DMD_human (x)   QQK.EAKVKLLTESVNSVIAQAPPVAQEALKKEL......ETLTTNYQWLCTRLNGKCKTLEE
DMD_human (x)   MRHSEDNPNQIRILAQTLTDGGVMDELINEELETFN........SRWRELHEEAVRRQKLLEQ
DMD_human (x)   LSEVKSEVEMVIKTGRQIVQKKQTENPKELDER......VTALKLHYNELGAKVTERKQQLEK
DMD_human (X)   EKQ.KVHLKSITEVGEALKTVLGKKETLVEDKLS.......LLNSNWIAVTSRAEEWLNLLLE
DMD_human (x)   RPK....VDSTRDQAANLMANRGDHCRKLVEPQIS......ELNHRFAAISHRIKTGKASIPL
DMD_human (x)   GQR.QTVVRTLNATGEEIIQQSSKTDASILQEKLG......SLNLRWQEVCKQLSDRKKRLEE
DMD_human (x)   PLR.QGILKQLNETGGPVLVSAPISPEEQDKLEN....KLKQTNLQWIKVSRALPEKQGEIEA
DMD_human (x)   APL.KENVSHVNDLARQLTTLGIQLSPYNLSTL.......EDLNTRWKLLQVAVEDRVRQLHE
ACTN3_human     PEA.DRERGAIMGIQGEIQKICQTYGLRPCSTNPYITLSPQDINTKWDMVRKLVPSRDQTLQE
```

Supplementary Figure S1. Alignments used to identify suitable mutation sites for this study. Where X is disease-causing mutation site and X is non-disease site. Sites marked X are the mutation sites selected for our model studies.

```
                  10        20        30        40        50
R15      ANKQQNFNTGIKDFDFWLSEVEALLASEDYGKDLASVNNLLKKHQLLEADISAHE
R16  KLNESHRLHQFFRDMDDEESWIKEKKLLVSSEDYGRDLTGVQNLRKKHKRLEAELAAHE
R17  RLEESLEYQQFVANVEEEEAWINEKMTLVASEDYGDTLAAIQGLLKKHEAFETDFTVHK
      * *      *         *  *   *  *****  *   * * ***   *     *
         <-------- A Helix ------->         <------ B Helix ----

         60        70        80        90        100       110
R15  DRLKDLNSQADSLMTSSAFDTSQVKDKRETINGRFQRIKSMAAARRAKLNE
R16  PAIQGVLDTGKKLSDDNTIGKEEIQQRLAQFVDHWKELKQLAAARGQRLEESLEYQQ
R17  DRVNDVCANGEDLIKKNNHHVENITAKMKGLKGKVSDLEKAAAQRKAKLDENSAFLQ
                                              ** *   * *     *
     --------------->    <---------- C Helix ---------->
```

Supplementary Figure S2. Model proteins used in this study. Alignments of Spectrin R15, R16 and R17 showing numbering used in this study and extensions added to ensure that domains are not artificially destabilized by being cut "too short". Residues in red are those residues that have been mutated in this study. The asterisks (*) indicate residues that are conserved between all three model proteins (including W17 and L104).

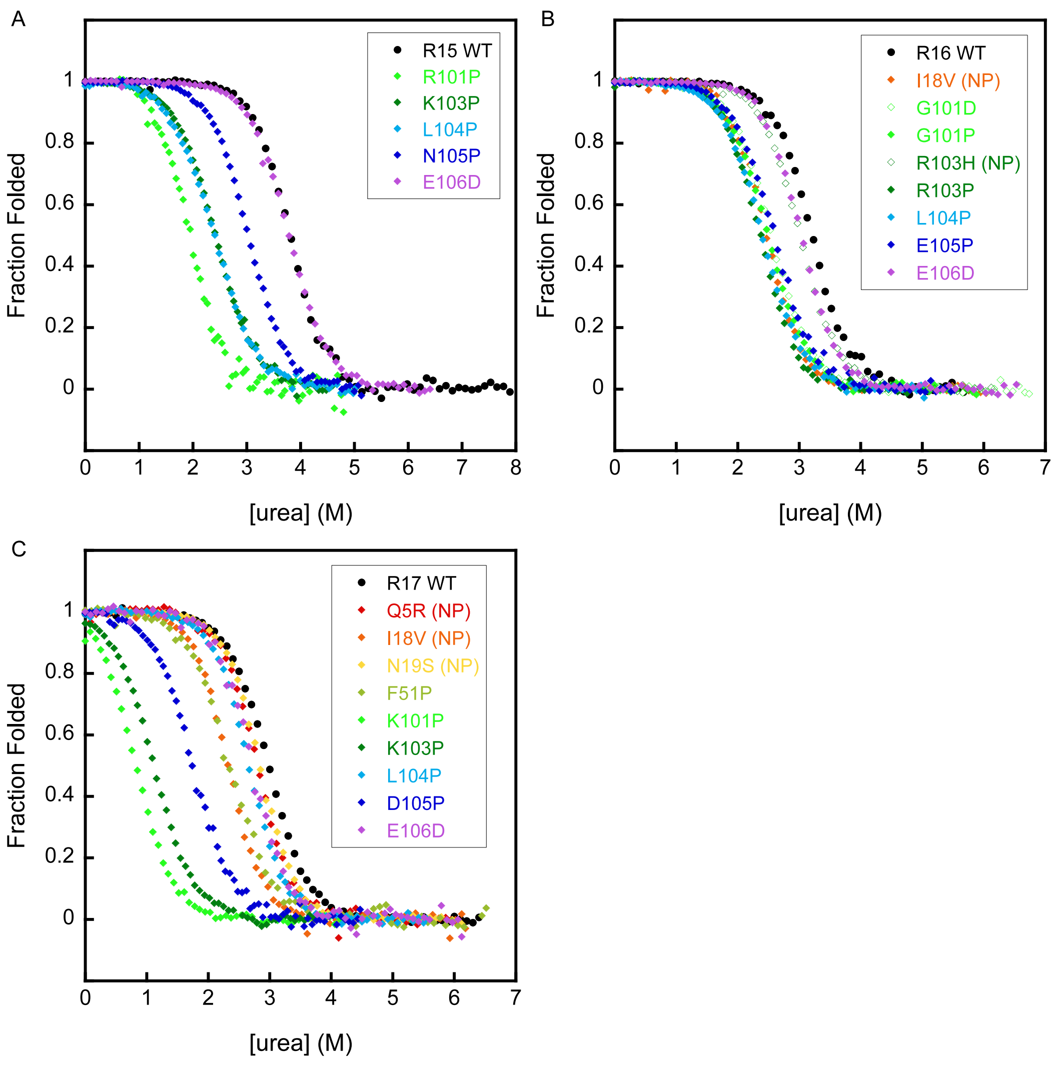

Supplementary Figure S3. Equilibrium denaturation curves of all single-domain proteins described in this study. (A) R15 WT and mutants, (B) R16 WT and mutants, (C) R17 WT and mutants. NP denotes non-pathogenic mutations.

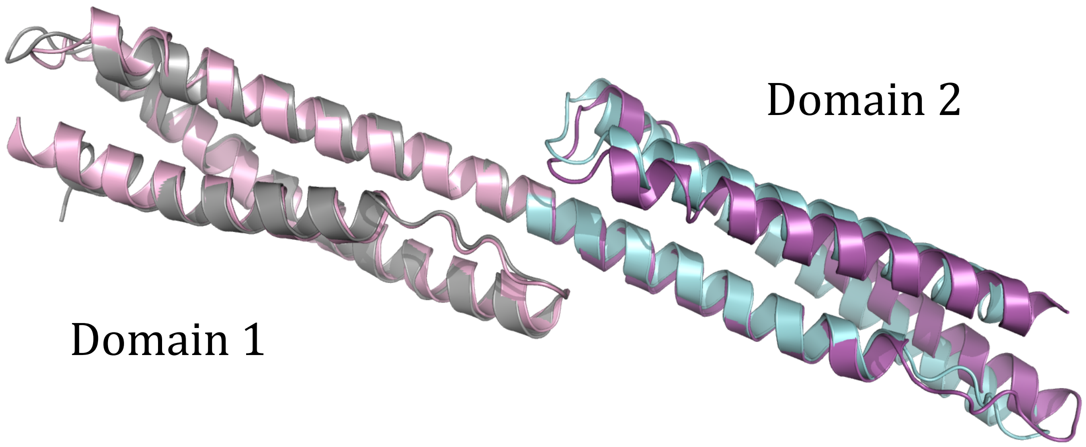

Supplementary Figure S4. The linking helix is conserved in spectrin repeats. Spectrin R1516 (grey/magenta) overlaid on spectrin R1617 (pink, light blue), showing conservation of structure in the linking helix and the rigid domain architecture.

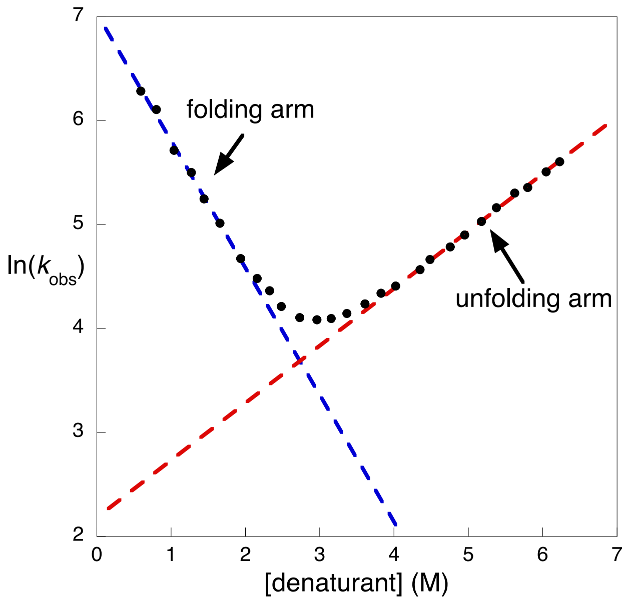

Figure S5. A chevron plot, showing folding and unfolding arms. Extrapolation of the folding and the unfolding arms (blue and red) allows determination of k_f and k_u at 0 M denaturant.

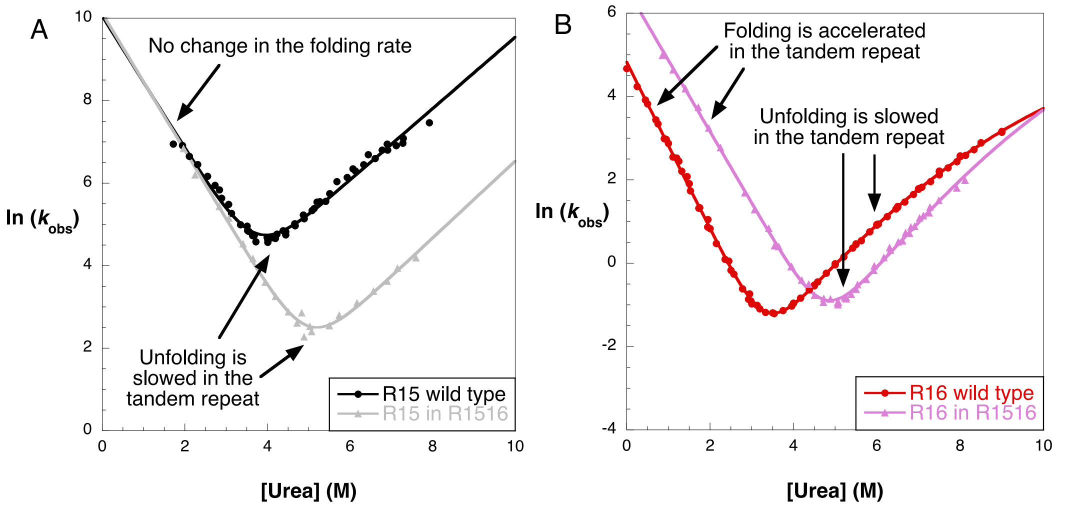

Figure S6. Comparison of the folding of R15 and R16, alone, and in R1516.

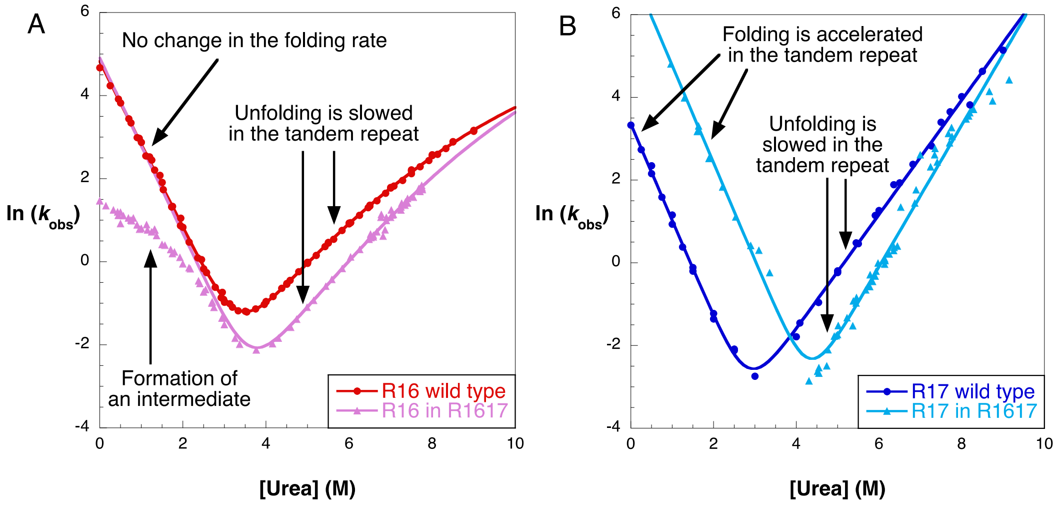

Figure S7. Comparison of the folding of R16 and R17, alone, and in R1617.

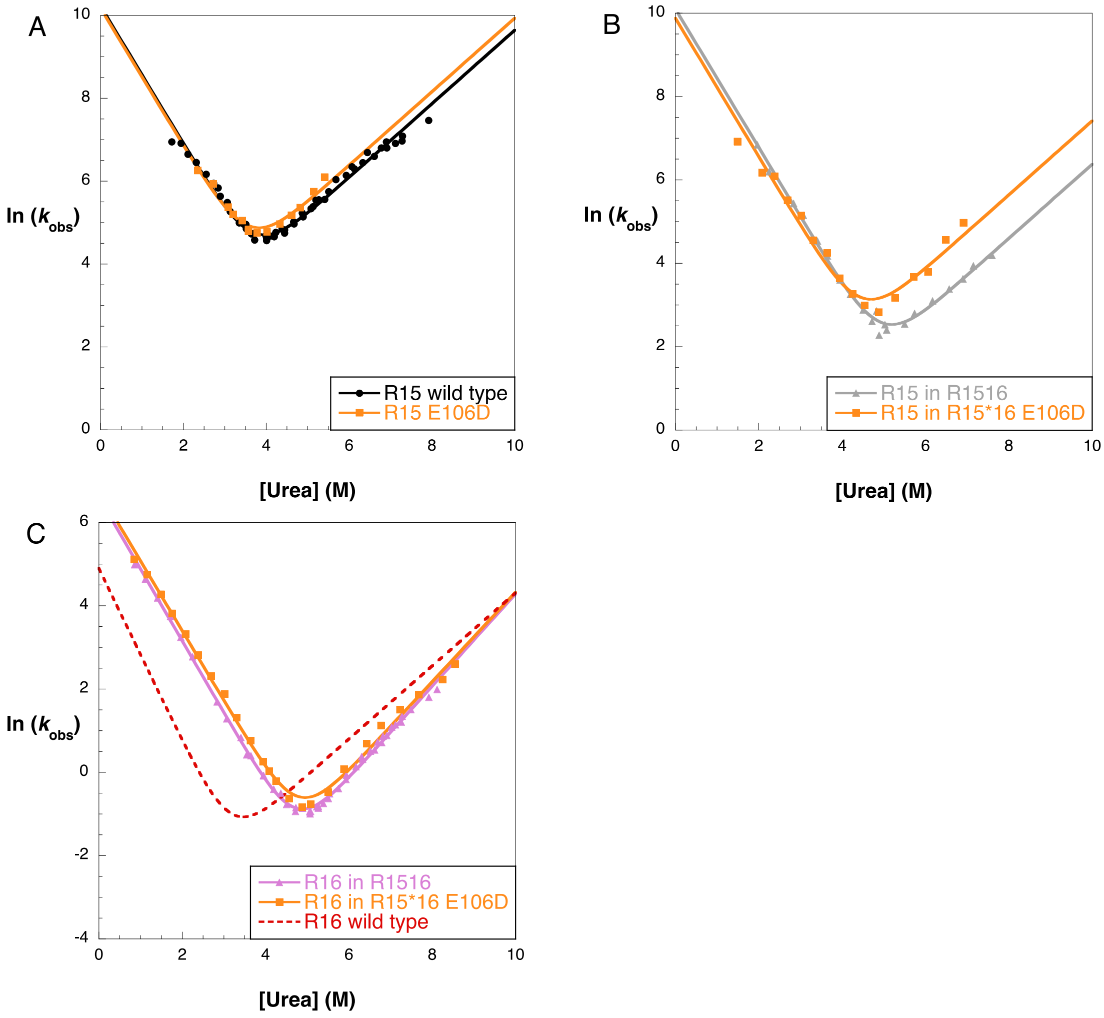

Figure S8. The mutation E106D does not disrupt inter-domain interactions in R1516.

(A) Single domains: R15 WT (black) and R15 E106D (orange) The folding rate is unaffected by the mutation. The mutation causes a small increase in the unfolding rate of R15. (B) R15 in R1516 WT (grey) and mutant (orange). The folding rate is the same, and again the mutation causes the protein to unfold a little more rapidly. However, the unfolding of the R15 domain is still very significantly slower than in the domain alone (compare to panel A) – R15 is still being stabilised by R16 in the mutant. (C) R16 in R1516 WT (magenta) and mutant (orange). The folding of R16 in the mutant R1516 is essentially identical to the R1516 WT form. The stability conferred by R15 is still present. (WT, single domain R16 is included for comparison, red dotted line). Compare this Figure with Figure 2, main text, where a mutation in R15 breaks all the stabilizing contacts with R16.

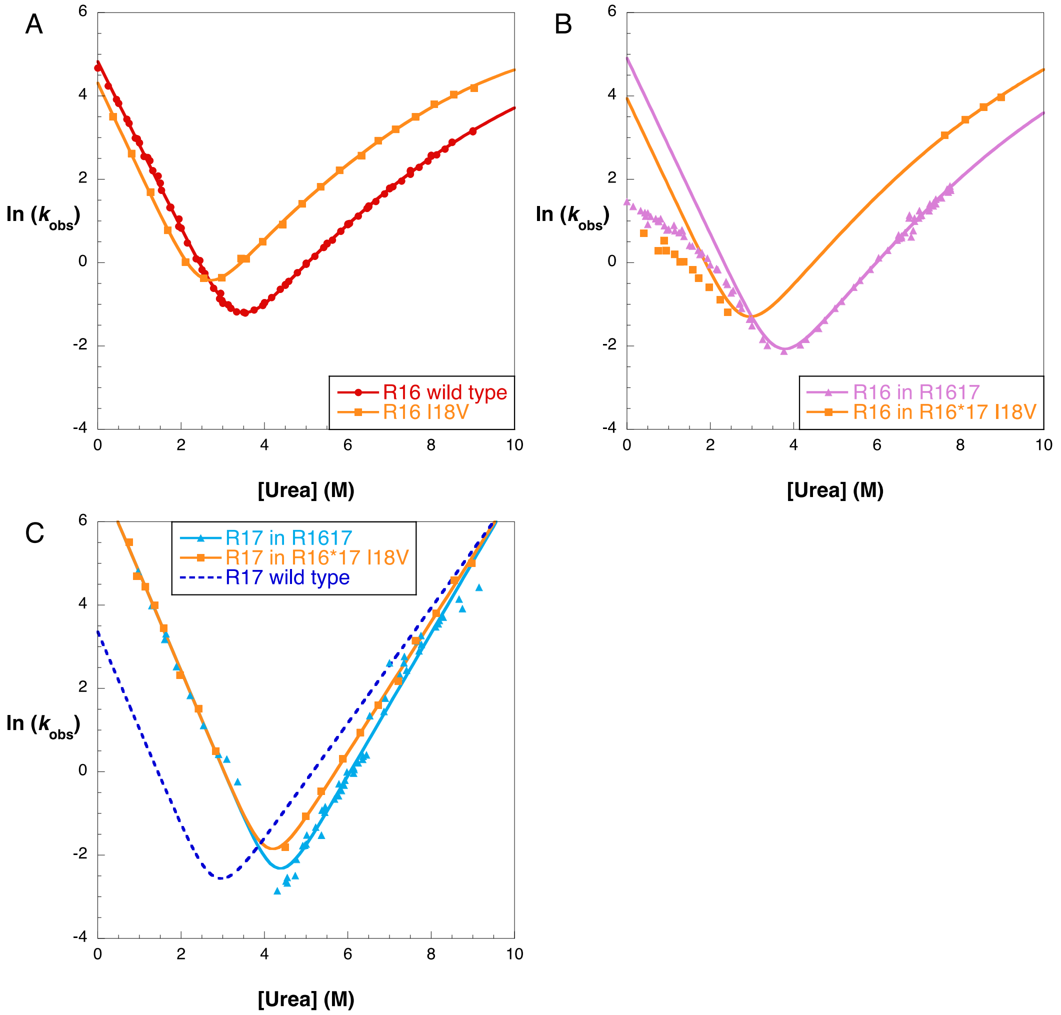

Figure S9. The mutation I18V does not disrupt inter-domain interactions in R1617.

(A) Single domains: R16 WT (red) and R16 I18V (orange). The folding rate is slowed very slightly by the mutation. The mutation causes a larger increase in the unfolding rate of R16. (B) R16 in R1617 WT (magenta) and mutant (orange). Again, the folding rate is slowed very slightly, and again the mutation causes the protein to unfold a more rapidly. However, the unfolding of the R16 domain is still slower than in the domain alone (compare to panel A) – R16 is still being stabilized by R17 in the mutant. (C) R17 in R1617 WT (light blue) and mutant (orange). The folding of R17 in the mutant R1617 is essentially identical to the WT form. The unfolding is slightly faster, but essentially we can conclude that the stability conferred by R16 is still present. (WT, single domain R17 is included for comparison, dark blue dotted line).

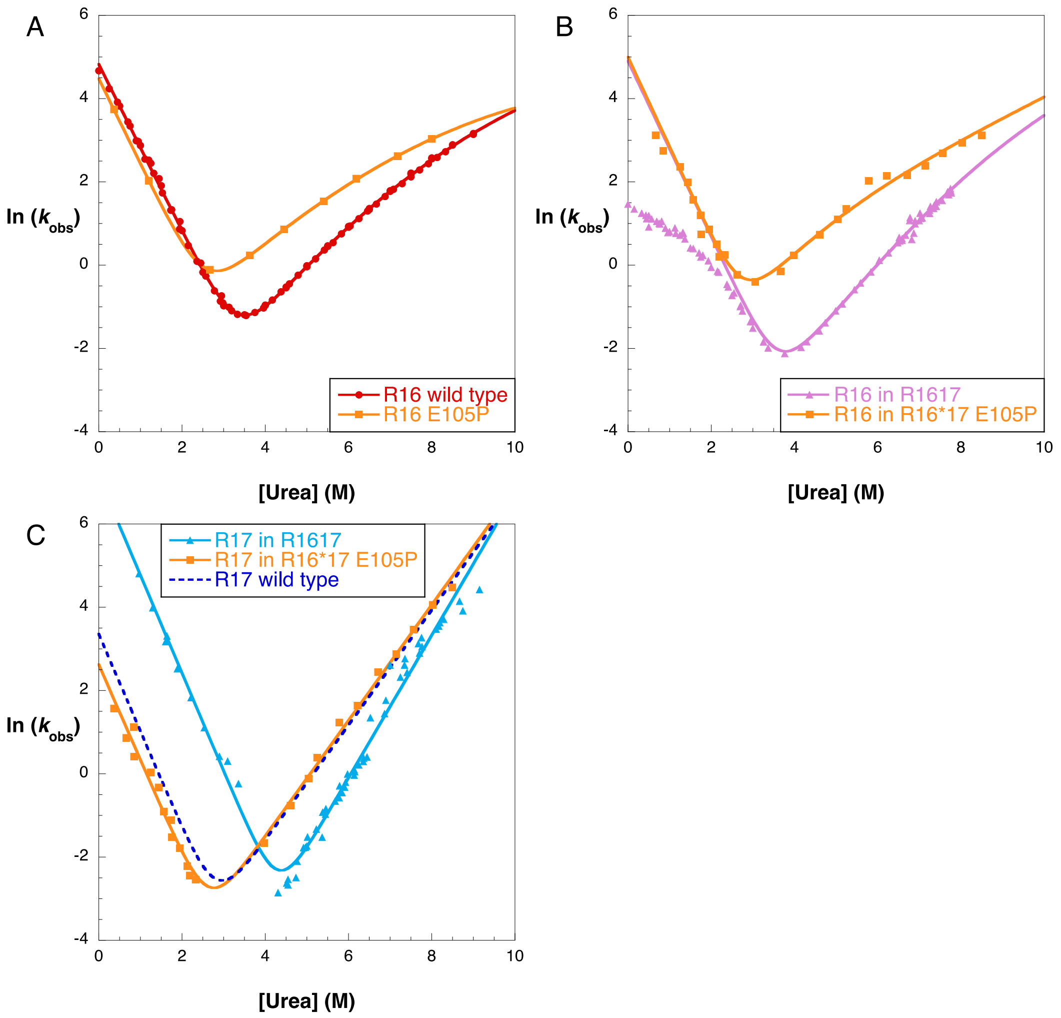

Figure S10. The mutation E105P destroys interactions between the domains.

(A) Single domains: R16 WT (red) and R16 E105P (orange). The folding rate is essentially unaffected by the mutation. The mutation causes an increase in the unfolding rate of R16. (B) R16 in R1617 WT (magenta) and mutant (orange). The mutation affects the R16 domain exactly as in the single domain protein (compare to panel A). The unfolding rate of the mutant is now significantly faster than R16WT in R1617. We do *not* see the characteristic roll over (formation of the intermediate). (C) R17 in R1617 WT (light blue) and mutant (orange). The effect is dramatic. The mutant protein folds much more slowly and unfolds much faster than WT. In fact it folds just like WT R17 single domain protein (included for comparison, dark blue dotted line). All the stabilizing interactions between R16 and R17 have been lost.
